# A Mobile Cloud-Based Parkinson’s Disease Assessment System for Home-Based Monitoring

**DOI:** 10.2196/mhealth.3956

**Published:** 2015-03-26

**Authors:** Di Pan, Rohit Dhall, Abraham Lieberman, Diana B Petitti

**Affiliations:** ^1^Biomedical Informatics DepartmentCollege of Health SolutionsArizona State UniversityScottsdale, AZUnited States; ^2^Muhammad Ali Parkinson CenterBarrow Neurological InstituteSt. Joseph’s Hospital and Medical CenterPhoenix, AZUnited States; ^3^Department of Biomedical InformaticsCollege of Medicine-PhoenixUniversity of ArizonaPhoenix, AZUnited States

**Keywords:** mHealth, Smartphone, Mobile App, Cloud application, Parkinson’s Disease, Home based monitoring, Telemedicine, Decision marking, Tremor, Gait difficulty

## Abstract

**Background:**

Parkinson’s disease (PD) is the most prevalent movement disorder of the central nervous system, and affects more than 6.3 million people in the world. The characteristic motor features include tremor, bradykinesia, rigidity, and impaired postural stability. Current therapy based on augmentation or replacement of dopamine is designed to improve patients’ motor performance but often leads to levodopa-induced adverse effects, such as dyskinesia and motor fluctuation. Clinicians must regularly monitor patients in order to identify these effects and other declines in motor function as soon as possible. Current clinical assessment for Parkinson’s is subjective and mostly conducted by brief observations made during patient visits. Changes in patients’ motor function between visits are hard to track and clinicians are not able to make the most informed decisions about the course of therapy without frequent visits. Frequent clinic visits increase the physical and economic burden on patients and their families.

**Objective:**

In this project, we sought to design, develop, and evaluate a prototype mobile cloud-based mHealth app, “PD Dr”, which collects quantitative and objective information about PD and would enable home-based assessment and monitoring of major PD symptoms.

**Methods:**

We designed and developed a mobile app on the Android platform to collect PD-related motion data using the smartphone 3D accelerometer and to send the data to a cloud service for storage, data processing, and PD symptoms severity estimation. To evaluate this system, data from the system were collected from 40 patients with PD and compared with experts’ rating on standardized rating scales.

**Results:**

The evaluation showed that PD Dr could effectively capture important motion features that differentiate PD severity and identify critical symptoms. For hand resting tremor detection, the sensitivity was .77 and accuracy was .82. For gait difficulty detection, the sensitivity was .89 and accuracy was .81. In PD severity estimation, the captured motion features also demonstrated strong correlation with PD severity stage, hand resting tremor severity, and gait difficulty. The system is simple to use, user friendly, and economically affordable.

**Conclusions:**

The key contribution of this study was building a mobile PD assessment and monitoring system to extend current PD assessment based in the clinic setting to the home-based environment. The results of this study proved feasibility and a promising future for utilizing mobile technology in PD management.

## Introduction

Parkinson’s disease (PD) is a progressive neurodegenerative disorder that affects more than 1 million US residents and about 3% of the population over the age of 65 in the world [1,2]. PD can cause significant physical and mental impairment and decreased quality of life [3]. When PD becomes clinically overt, tremor, bradykinesia, rigidity, and impaired postural stability are the four cardinal motor signs [4]. Patients may also suffer from shuffling of gait, freezing of gait, and dystonia [5]. Current PD management requires regular assessment and close monitoring of symptoms in order to adjust medication dosage and frequency, especially when motor complications of therapy appear. Assessment is generally conducted using brief observations by the physician during a patient visit. Assessment in this clinical setting is subjective and it is difficult to keep track of decline and improvement of symptoms between clinic visits [6]. Closer monitoring of PD symptoms has the potential to permit more informed decisions about therapy. Achieving closer monitoring with more frequent clinic visits increases the physical and economic burden for PD patients and their families [7].

Given the characteristics of Parkinson’s disease and its challenges on disease management, designing ambulatory tools for remote monitoring of PD patients has also attracted a lot of attention recently [8-13]. The eddy-current detector, commercial portable multichannel recorder, and wearable sensor have been used to measure hand tremor [14-16]. In a recent study, Rodriguez-Molinero et al used a portable inertial sensor to detect motor fluctuations (on-off) in PD patients, and the result showed very high sensitivity and specificity [17]. In terms of gait, there are three primary measurements of gait: (1) force-based measurement, (2) angular rate measurement, and (3) accelerometer measurement [18]. Several accelerometer-based measurement systems for ambulatory monitoring of gait-related symptoms in PD have been reported in freezing of gait detection, posture and walking speed estimation, and fall risk estimation [3,19-21]. Salarian et al used body-attached gyroscopes to estimate gait features and physical activities related to PD. However, their study did not report any result about how to use the estimated features to detect and estimate PD severity [22,23]. Patel et al, in Harvard medical school, used wearable accelerometers to evaluate motor complications on persons with PD, and attempted to predicate the clinicians’ estimates of disease symptoms severity [8,24,25]. But their approach needed patients to attach several sensors at different locations and also required a separate control module to transmit and store data. The requirement of these extra settings puts an additional burden on users and decreases the usability of the system.

With the rapid development of sensor technology, cloud computing, and ubiquitous access to the Internet from mobile devices, eHealth and mobile health have spurred the development of telemedical systems that monitor vital signs and physiological signals, including electrocardiograms and electromyography, with several being marketed [8,26-28]. With the integrated sensors in modern smartphones becoming more powerful and cheaper, the feasibility and accuracy of using smartphones to measure various movement-related metrics have attracted a lot of research interest. Fontecha et al recently reported using tri-axels accelerometers in smartphones to assess frailty in elderly people [29]. Liddle et al used the global positioning system (GPS) sensor in smartphones to evaluate lifespace of people with PD [30]. Galan-Mercant et al utilized the accelerometer and gyroscope to measure sit-to-stand posture transition in elderly persons [31]. Recently, Apple unveiled its plan to embark on health care by releasing HealthKit APIs in iOS 8 in June 2014. These provide efficient tools and an interface for developers to develop apps to access, manage, and transfer information about health and well-being with a wearable device. With these technology evolutions, it is feasible and very promising to extend PD monitoring from intermittent clinic-based assessment to the home-based environment by leveraging current mobile device and powerful cloud computing.

In this project, we designed, developed, and evaluated a mobile cloud-based app, “PD Dr”, for Parkinson’s disease home-based monitoring and assessment. PD Dr assesses users’ motor performance by capturing motion data using the embedded 3D accelerometer of a smartphone, identifying key symptoms, and estimating symptom severity based on this captured data. In this paper, we first describe system architecture, design, and development. We then present the initial test results. We end by discussing design considerations, potential limitations, and future directions.

## Methods

### System Description and Architecture

**Figure 1:**
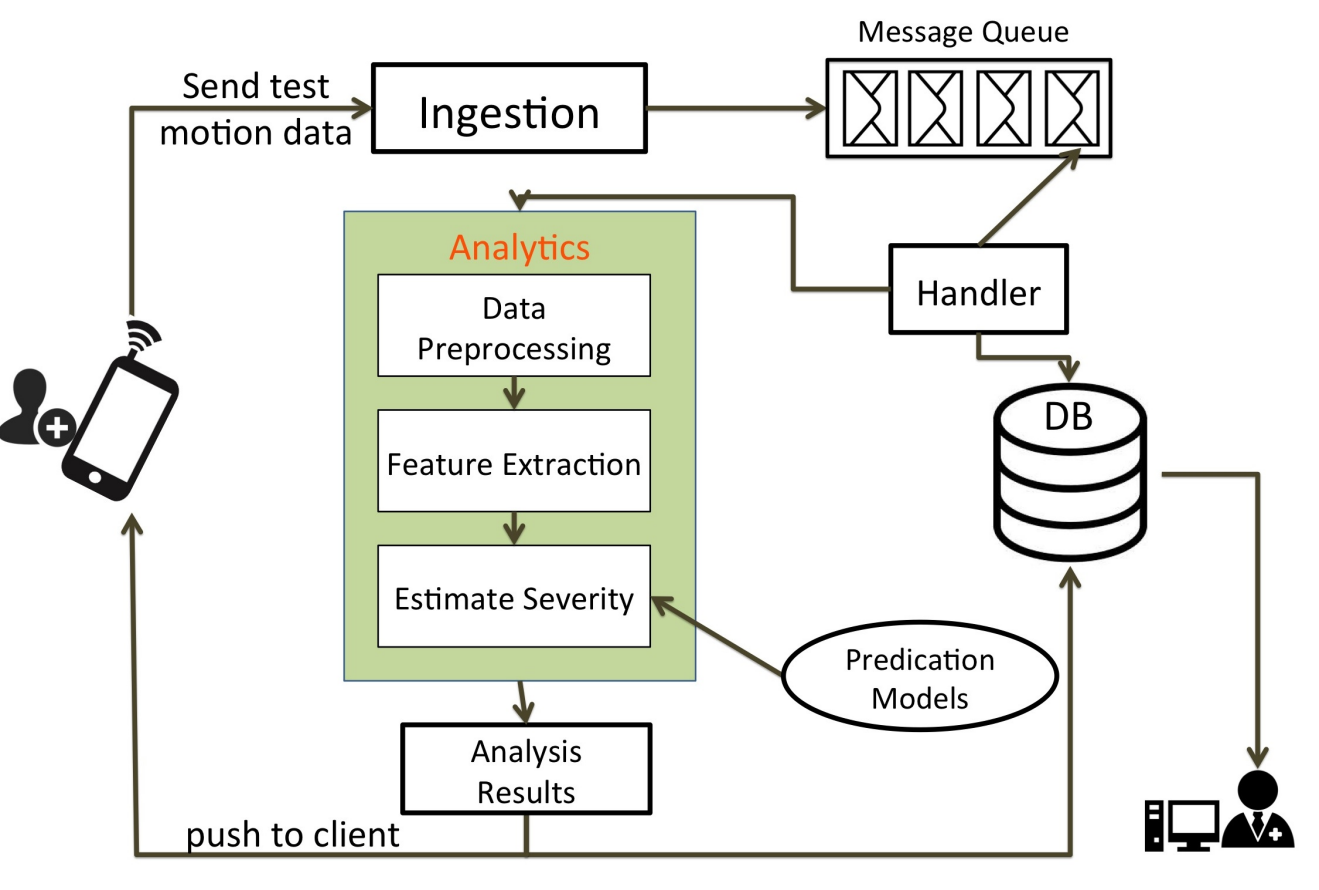
System architecture and data communication flow.

PD Dr is a mobile cloud app that utilizes the 3D accelerometer in a smartphone to collect data on hand tremor and walking motion, and utilize high computing performance and cloud service storage to analyze disease severity and monitor disease progression. The system is composed of two parts: a mobile app on a smartphone for motion data collection and user interaction, and a cloud service that processes the motion data and stores results. Patients use the client app to test their own performance, send the motion data to a cloud service, and receive evaluation results back from the cloud. Figure 1 displays the overall system architecture and data flow.

The mobile app on the smartphone captures patients’ movement accelerations while they conduct a motor performance task. The smartphone is mounted on the back of the hand or ankle of the patient with a strap, and instructions on screen guide them through the motor performance task. Data captured by the 3D accelerometer embedded in the smartphone are temporarily stored locally on the device. Once a task is finished, captured motion data and metadata are sent to a cloud service. The cloud application processes received data through a data pipeline that estimates disease severity and the estimate is sent back as a report to the patient’s smartphone. All motion data and analysis results are stored in a cloud database that is used for tracking disease history.

In PD Dr, users’ privacy and data security are assured at three levels: at the mobile app level, the data transmission level, and the data storage level. In the mobile app, user log-in is required in order to perform the test and browse test history. All application data stored on the local mobile device are encrypted; the data are deleted from the device after sending to the cloud server. The mobile app does not store or display any patient identity information, in accordance with Health Insurance Portability and Accountability Act (HIPAA) regulations. At the data transmission level, data are encrypted and transmitted through secure hypertext transfer protocol (https). At the server level, data are stored in the database in encrypted format and only an authorized database administrator has access.

### Motor Performance Test Design

**Figure 2:**
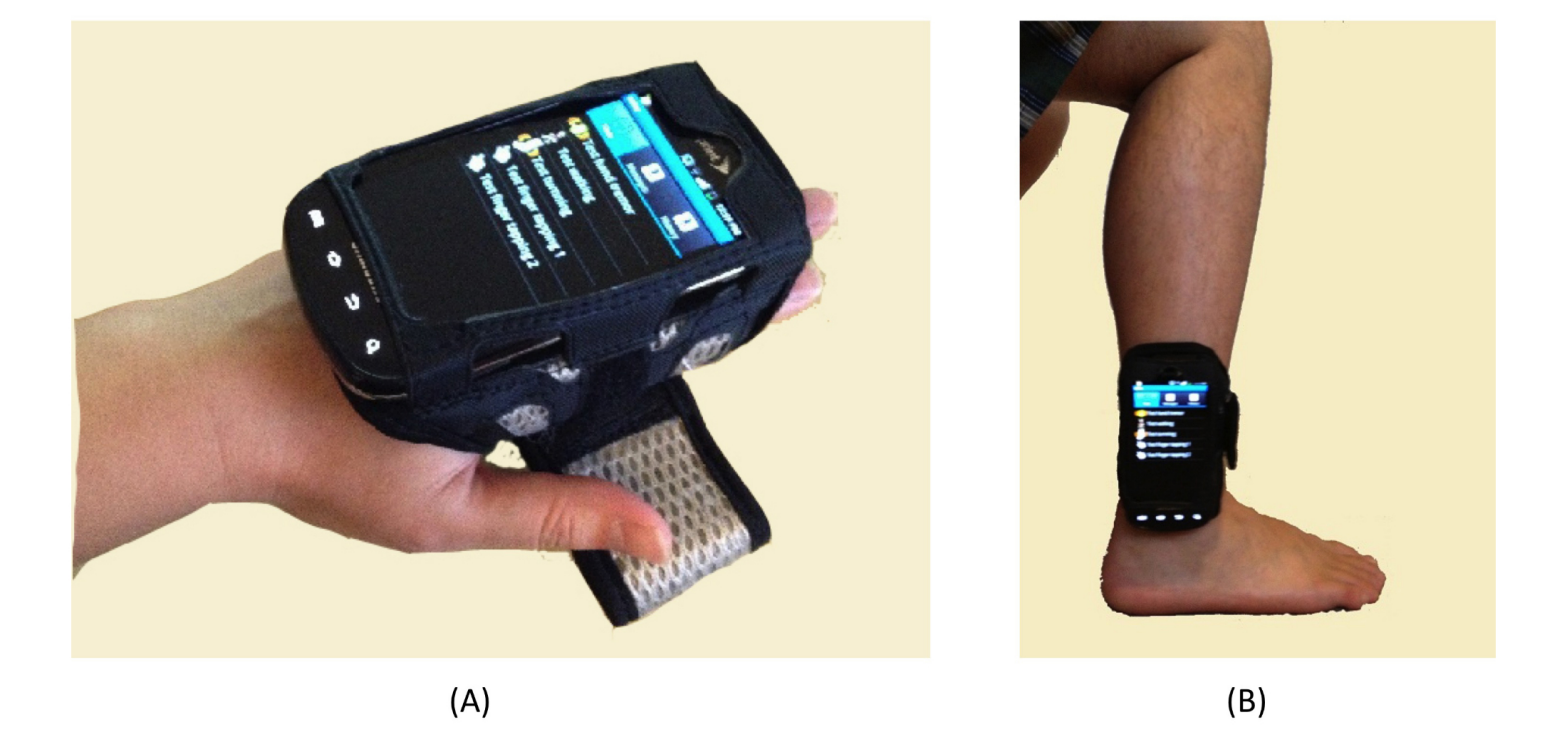
Smartphone and motor test application in hand tremor test (A) and walking & turning test (B).

PD Dr measures motion in three motor performance tasks: hand resting tremor, walking, and turning. Selection of these three tasks was based on previous studies that found a strong association of the three motor performances with PD severity. Hand resting tremor is a typical symptom that is found in most early stage PD patients [32]. A strong association exists between PD lower body motor disabilities and walking/turning performance [33]. Table 1 provides a detailed explanation of the motor tasks. Figure 2 depicts the mount positions of the smartphone in the hand resting tremor, walking, and turning tasks.

**Table 1:** Designed motor performance test on PD Dr app.

Test name	Test description	Captured data description
Hand tremor	User attaches the smartphone to the back of the hand and leaves the hand hanging for 20 seconds.	Translational acceleration rate at X, Y, and Z directions and angular acceleration rate at pitch, roll, and yaw directions.
Walking	User attaches the smartphone to the ankle of one leg and walks 25 feet.	Translational acceleration rate at X, Y, and Z directions and rotation matrix of smartphone with time change.
Turning	User attaches the smartphone to the pivot leg in turning 360°.	Angular acceleration rate at pitch, roll, and yaw directions with time change.

### Mobile App: Data Acquisition and Communication

The client mobile app was developed on the Android 4.0 platform. The sampling rate of the accelerometer was set to 100 Hz. Since hand tremor acceleration is lower than 20 Hz [34,35], and walking and turning acceleration is between 2~5 Hz [36], 100 Hz sampling rate is sufficient to capture PD-related motion features. Because the device might not have Internet access in certain conditions, an internal relational database SQLite was utilized as temporary local data storage to store captured motion data. To accommodate situations where neither a wireless network nor mobile phone network is available, acquired motion data can be temporarily stored in the internal database and then sent to the server side when a network is available. All data was marshaled into XML format and then encrypted using Advanced Encryption Standard (AES) algorithm for transmitting to the cloud server through the Internet [37].

**Figure 3:**
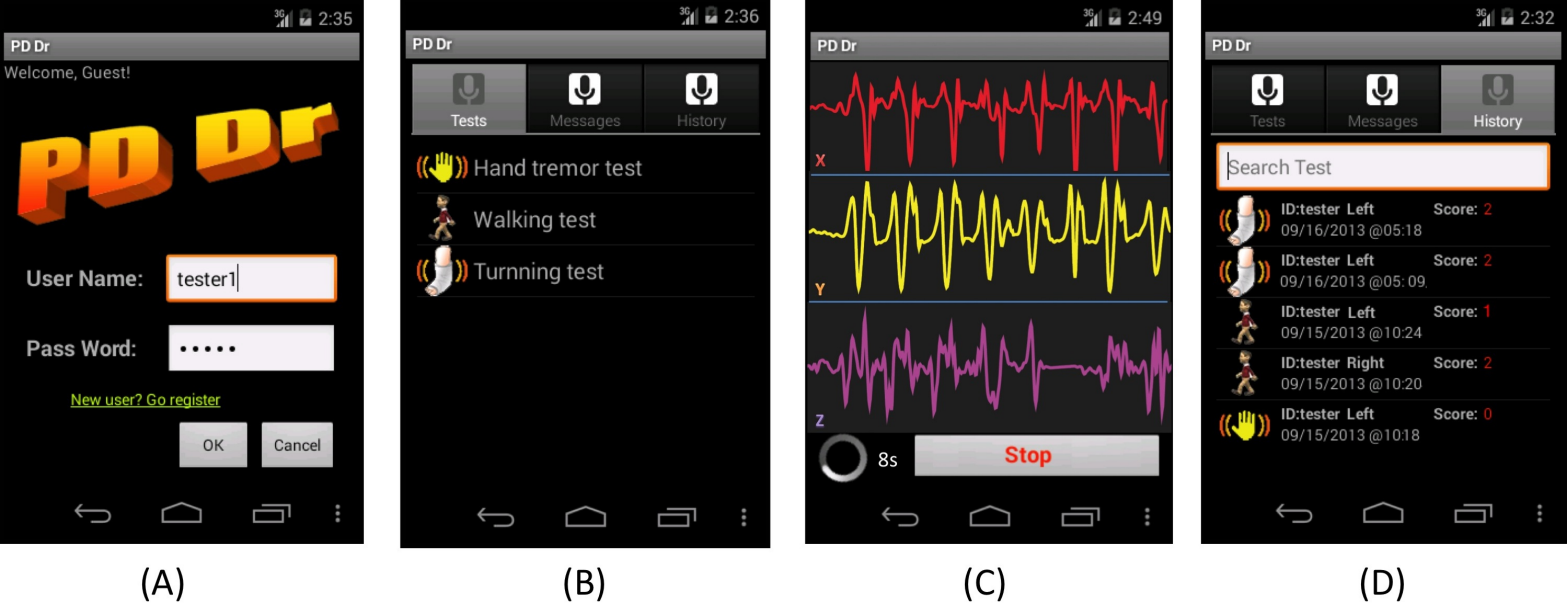
Screenshots of PD Dr app: (A) User account log-in, (B) Motor test list, (C) Data collection during test, and (D) Test history and evaluation result.

The mobile app is made up of four function modules, shown in Figure 3. The first module is user log-in and account verification. Users must first log in with their credentials to perform the tests. See Figure 3 (A). The second module, the motor performance test module, lists the three tests for user selection. See Figure 3 (B). After the user selects a test, the test view appears and displays step-by-step instructions on how to conduct the test. As the user performs the test, captured motion data are displayed on the smartphone screen in real time; the data are saved on the smartphone once the test is completed, as shown in Figure 3 (C). The third module is a communication module, which integrates with short message service (SMS) and email. Users can send questions or receive medical recommendations from the medical care facility server. The fourth module is the test history management module. It is composed of a list view and search field. Users can browse test history chronologically or search a specific test. Once a target test record is found, the user can click on a test record to review details, send it to a server, or delete it from the smartphone. See Figure 3 (D).

### Cloud Service: Data Processing and Decision Making

**Figure 4:**
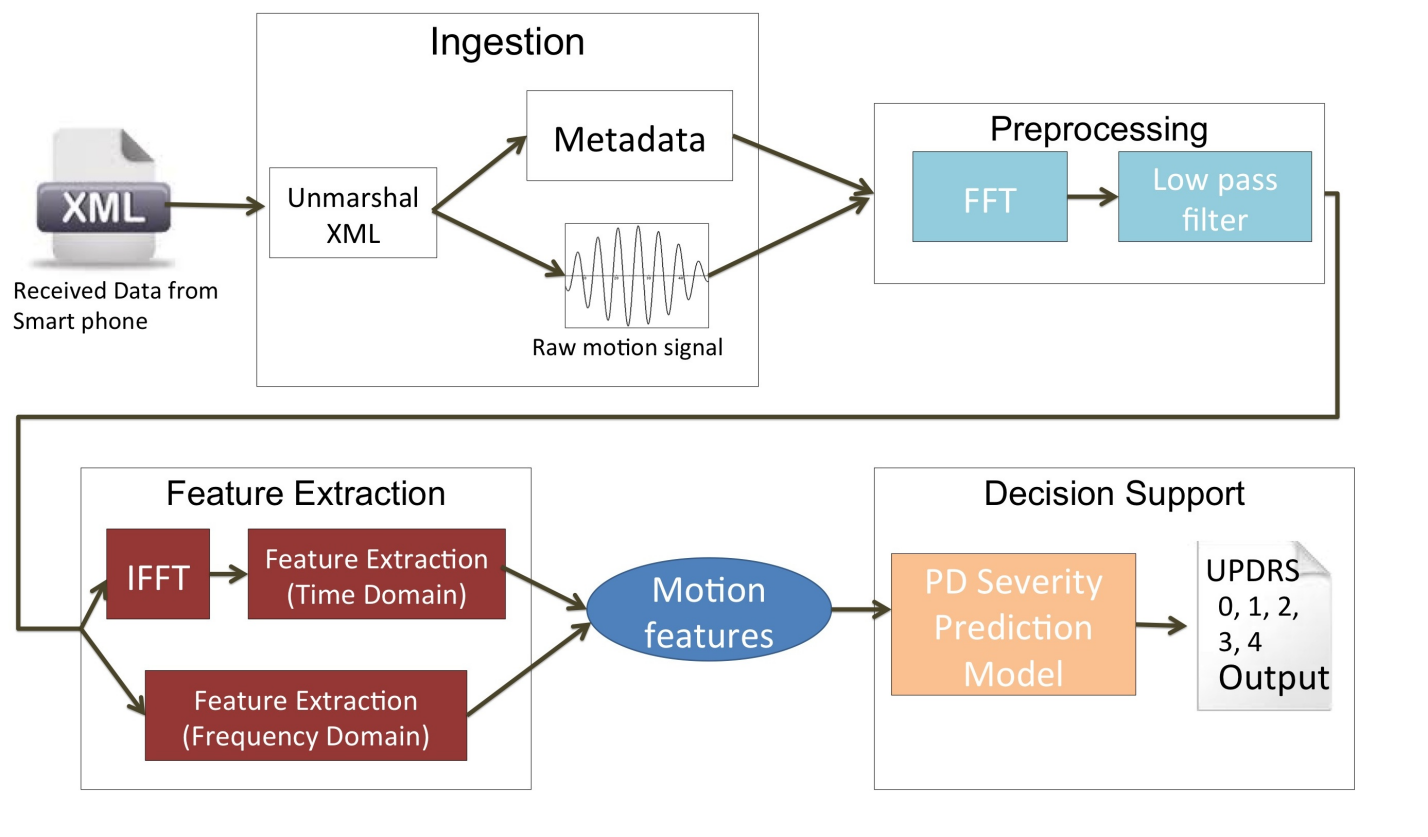
Cloud data processing pipeline.

The cloud service is composed of three components: (1) a data processing pipeline, (2) disease severity predictive models, and (3) database storage. The framework of server side components is depicted in Figure 4. Motion data are first received by the ingestion module in encrypted XML. For each received test record, the data ingestion module decrypts and parses out the raw motion data and associated metadata, which consists of sampling rate, time duration, date, test type and user ID. The ingestion module then puts the test record into a message queue for asynchronized handlers to process. Each asynchronized handler pulls a message from the message queue, then sends the data to the database, where it is analyzed through the data processing pipeline. The main reason to use a message queue and asynchronized handler to process data is to increase system scalability and decouple the system components. The data processing pipeline executes a series signal processing steps and data analysis steps. The pipeline first filters out noise through low pass filter and then calibrates acceleration to zero baseline.This is followed by analytics to extract PD-related motion features using several signal processing and motion pattern extraction algorithms that were introduced in previous studies [38-40]. Tables 2 and 3 provide a detailed description of extracted motion features for hand resting tremor, walking, and turning. The extracted motion features are then fed into a decision support module that uses the information to estimate disease severity based on Part III of the Unified Parkinson’s Disease Rating Scale (UPDRS) and disease stage using the Hoehn&Yahr scores [41]. All of the cloud components, including the motion ingestion module and data processing pipeline, were home-developed using Java programming language and were built on the Spring web framework.

The decision support module was designed to detect critical movement disability symptoms and to estimate PD severity. In this prototype, the decision support module could provide severity estimation of hand resting tremor and gait difficulty, as well as PD disease stage estimation. We utilized a subset of data mining techniques to assess whether hand resting tremor and gait difficulty are characterized by specific patterns and to estimate disease severity from the hand resting tremor motion features and gait features respectively. Input features for hand resting tremor and gait difficulty were extracted motion features, as described in Tables 2 and 3. The motion features of hand resting tremor were selected based on previous study results on characteristics of resting tremor, as well as experts’ opinions [34,35,42]. Two binary classification models were trained using Support Vector Machine (SVM), to detect gait difficulty and hand resting tremor [43,44]. For estimating symptom severity, we built three regression models to estimate disease stage (Hoehn&Yahr score from 1-5), hand resting tremor UPDRS score, and gait difficulty UPDRS score, using the Lasso regression approach [41,45,46].

**Table 2:** Extracted hand resting tremor motion features.

Tremor features	Description
PF4_6	The power of the motion data between 4 and 6 Hz.
%PF4_6	Fraction of power of motion data between 4 and 6 Hz.
PR	Power ratio of the motion data in 3.5~15 Hz to 0.15~3.5 Hz frequency components
PF0_20	The total power of motion data from 0~20 Hz
PEAK_POWER	The peak power value of hand resting tremor motion data.
AVG_ACC	The average acceleration of motion of hand resting tremor.

**Table 3:** Extracted gait motion features.

Gait features		Description
**Walking Straight Task**		
	CT (s)	Average gait cycle time.
	SL (m)	Average stride length.
	SP (m/s)	Average walking speed.
	AVG_ACC (m/s^2^)	Average acceleration during walking.
**Turning 360** ^o^		
	NUM_TURN	The number of steps used to finish turning 360^o^.
	TURN_SP	The speed of turning 360^o^, calculated by 360^o^ / time.

### System Evaluation

PD Dr was tested and evaluated by recruiting patients with PD to use this system. The involvement of PD patients in the project was approved by the St. Joseph’s Hospital and Medical Center (SJHMC) Institutional Review Board. Patients who received UPDRS-based evaluations as part of their regular clinic visits were invited to participate in this study, except mentally incompetent individuals and those with conditions that increased their vulnerability. The study instructions and consent forms were given to patients and explained by the principal investigator. If patients agreed to participate in this study, they signed the consent forms, which were collected by the principal investigator before testing. Participation was voluntary and participants could withdraw at any time during the study. We recruited 40 patients with a diagnosis of PD who had motor symptoms from among outpatients seen in the Muhammad Ali Parkinson Center. Hand resting tremor, walking, and turning motion data were collected through PD Dr. Two movement disorder experts also evaluated the severity of PD symptoms and disease stages of these 40 test subjects. Their expert ratings and evaluation of disease stage were treated as ground truth in training the prediction models used in decision support module. Collected motion data were analyzed and validated against the experts’ evaluation of disease severity.

## Results

### Test Subject Description

Table 4 shows the general characteristics of the 40 PD patients in this study. Among these 40 patients, 5 were female and 35 were male. Ages of the participants ranged from 44 to 84 years, and the average age was 68.5 years old (SD 9.5). Among all subjects, 16 were in early disease stage (disease duration less than 6 years), and 24 subjects were in late disease stage (disease duration more than 6 years). The average Hoehn&Yahr stage was 2.4 (SD 0.8). There were 6 subjects at stage 1, 13 subjects at stage 2, 12 subjects at stage 3, and 9 subjects at stage 4. No subjects at stage 5 were recruited in this study. Among these 40 subjects, 9 subjects had freezing of gait (FoG), 11 subjects had gait difficulty other than FoG, 19 subjects had postural instability, and 5 subjects reported having falls on a weekly basis.

**Table 4:** Patients’ general characteristics and UPDRS^a^ scores (N=40).

		Mean (SD) or number	Range	Median (Q1-3)
Age (years)		68.5 (9.5)	44-84	70 (63-74)
Disease duration (years)		6.6 (4.0)	0-19	6 (4-8)
Hoehn&Yahr stage		2.4 (0.8)	1-4	2 (2-3)
**Presence of motor related disabilities (Yes/No)**				
	Bradykinesia	10/30	N/A	N/A
	Freezing of gait (FoG)	9/31	N/A	N/A
	Gait difficulty	11/29	N/A	N/A
	Postural stability problem	19/21	N/A	N/A
	Falls	5/35	N/A	N/A

^a^UPDRS: Unified Parkinson’s Disease Rating Scale

### Hand Resting Tremor

**Figure 5:**
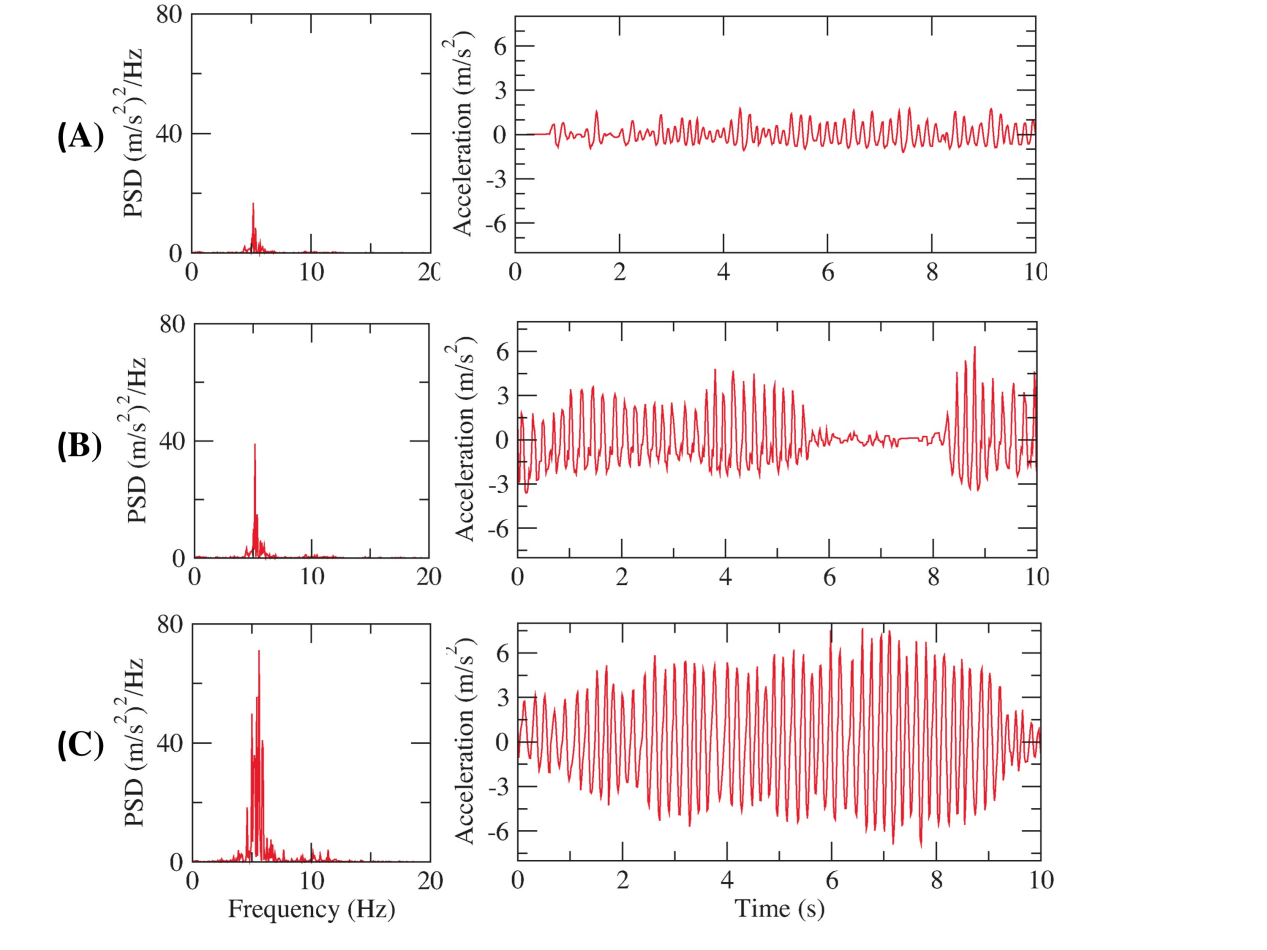
Acceleration waveform and power spectrum density (PSD) plot of hand resting tremor of different severity: (A) Mild hand resting tremor, (B) Moderate hand resting tremor, and (C) Severe hand resting tremor.

Collected hand resting tremor motion data were analyzed and compared between subjects with different disease severities. Figure 5 shows hand resting tremor motion data for three subjects with UPDRS tremor score (UPDRS III Item 20) ranging from 1~3, which maps to mild, moderate, and severe. Power spectrum density (PSD) plots are shown in the left column, and corresponding accelerations are shown in the right column. The patient with mild hand tremor, Figure 5 (A), shows average acceleration of hand motion of 0.47 m/s^2^. The PSD plot shows power spectrum mainly dominated around 5 Hz and the peak power was 16.8 (m/s^2^)^2^/Hz. For the patient with intermediate hand tremor, Figure 5 (B), the peak acceleration was at 6.3 m/s^2^, and peak PSD was at 39.01 (m/s^2^)^2^/Hz. The patient at intermediate severity shows intermittent hand tremor, with average acceleration at 1.7 m/s^2^ when hand tremor appears. Figure 5 (C) shows the motion data of a patient with severe hand resting tremor. It can be observed that continuous large acceleration of tremor movement appeared at 3~5 m/s^2^, with peak PSD at 71.2 (m/s^2^)^2^/Hz.

From the above comparison, the results show that acceleration of hand tremor from PD Dr can demonstrate distinct quantitative characteristics according to change of severity. Moreover, our results are congruent with the observation rest tremor in PD predominates in 4~6 Hz [34]. The acceleration and PSD analyses demonstrate that hand tremor motion data collected from PD Dr can effectively capture motion features to characterize tremor, and provide a quantitative measurement for hand resting tremor.

### Gait Difficulty

**Figure 6:**
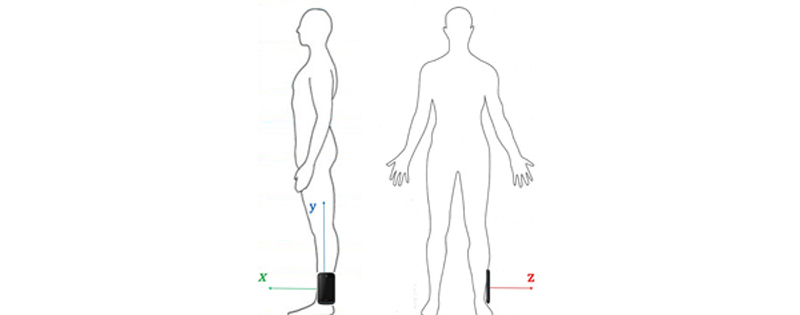
The mount position of the smartphone in walking and turning task.

**Figure 7:**
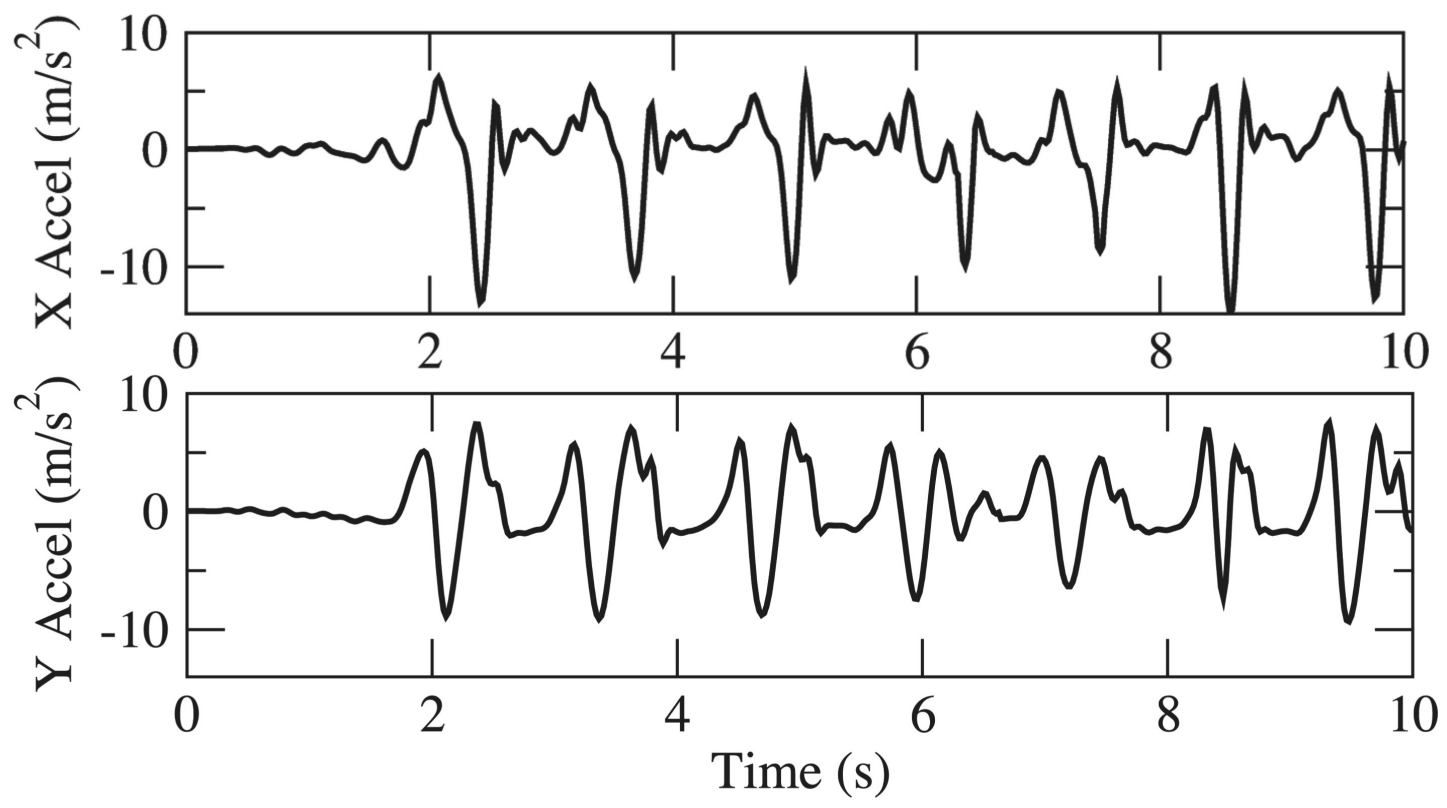
Acceleration in walking, Unified Parkinson’s Disease Rating Scale (UPDRS) gait score = 0.

**Figure 8:**
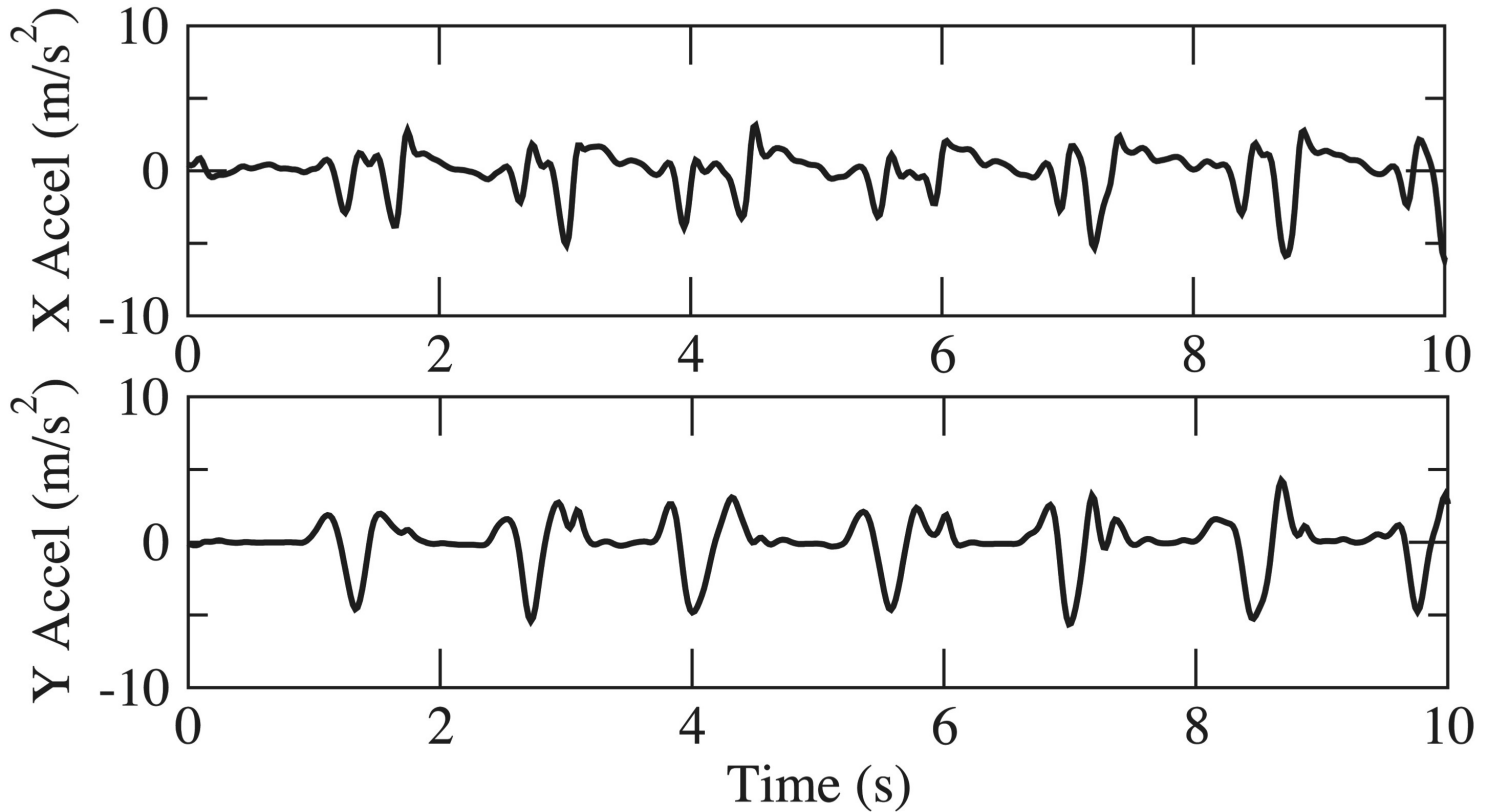
Acceleration in walking, Unified Parkinson’s Disease Rating Scale (UPDRS) gait score = 1.

**Figure 9:**
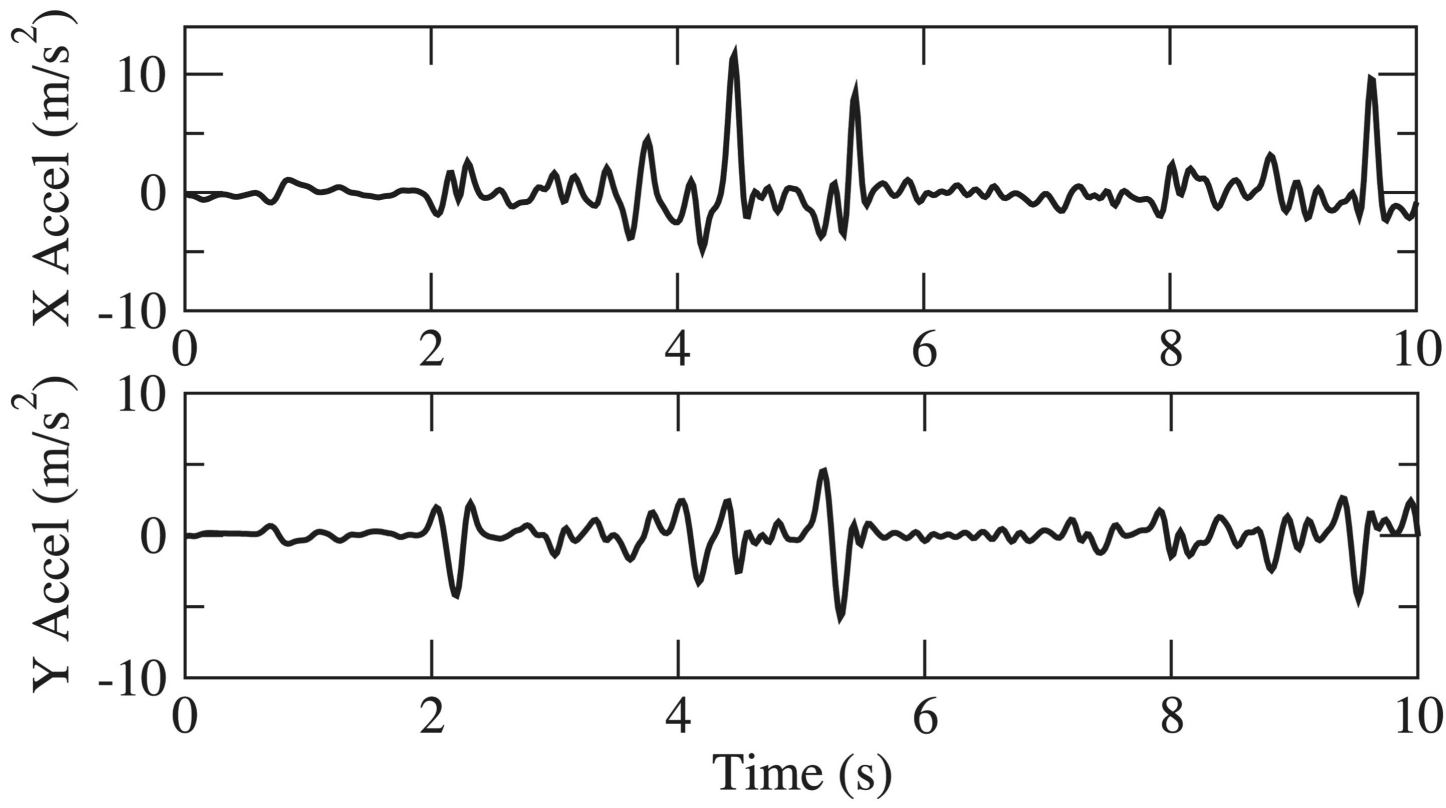
Acceleration in walking, Unified Parkinson’s Disease Rating Scale (UPDRS) gait score = 2.

**Figure 10:**
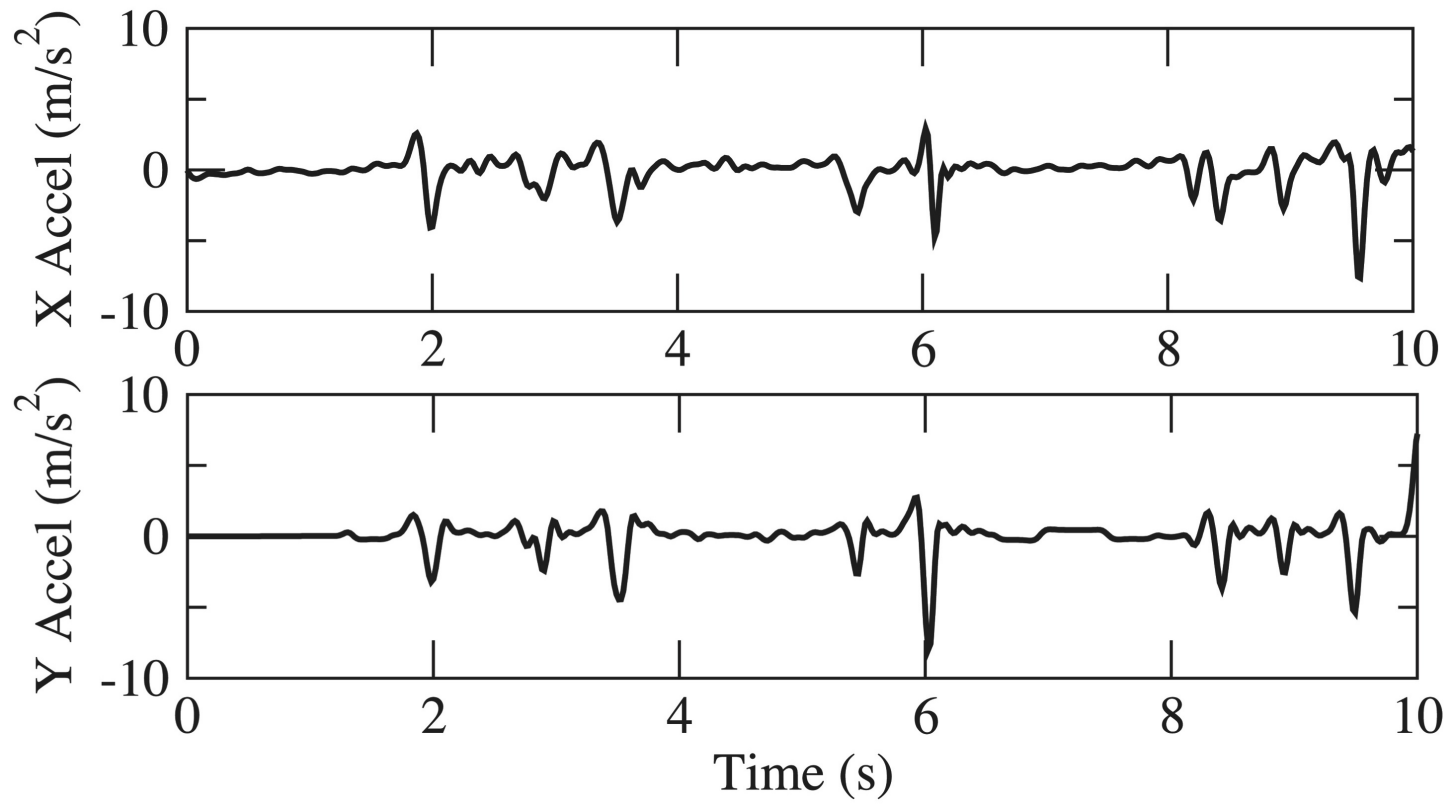
Acceleration in walking, Unified Parkinson’s Disease Rating Scale (UPDRS) gait score = 3.

Over the course of PD, motor impairment in the lower body can substantially impair walking, balance, and postural stability, putting patients at risk for falling. PD Dr captures lower body movement from measurements of 3D acceleration of the ankle during walking and turning, as shown in Figure 6. Sample gait motion data from four test subjects with UPDRS gait score (UPDRS III Item 29) at 0, 1, 2, and 3 (according to ascending of severity) respectively, are shown in Figures 7-10. Figure 7 shows the gait acceleration waveform from a subject with UPDRS gait score of 0, with no gait difficulty observed. The gait profile exhibits the repetitive pattern of gait cycles: each gait cycle is composed of a swing phase and a stance phrase. Peak acceleration in each gait cycle generally remains consistent. Positive peak acceleration is up to 8 m/s^2^, and the negative peak acceleration is up to 12 m/s^2^. As severity of gait impairment increases, gait patterns in each cycle become more volatile and peak accelerations decrease substantially. In Figure 8, from a subject with UPDRS gait score of 1, repeating gait cycles can still be observed and the acceleration in each gait cycle is still stable. However, the peak acceleration decreases substantially to less than 5 m/s^2^. When UPDRS gait difficulty increases to 2, shown in Figure 9, regular gait patterns disappear. The duration of gait cycles shows large variation, and the swing and stance phases become obscure. Accelerations of ankle movement also show large fluctuations. Figure 10 shows the data from a test subject who was diagnosed with FoG. It can be seen that the gait cycle is discontinuous, and there is a long gap from 3.6 s to 5.3 s, and from 6.2 s to 8.2 s; during those two periods, the test subject was unable to move.

To further evaluate the performance of PD Dr in capturing key motion characteristics of PD symptoms, gait cycle time (CT), stride length (SL), walking speed (SP), and average acceleration (ACC) of all 40 PD patients were extracted. These four features were compared between patients without gait difficulty (UPDRS gait difficulty score <2) and patients with gait difficulty (UPDRS gait difficulty score ≥2). The statistical significance of differences between these two groups was tested based on the *t* test. Table 5 shows the result of this analysis. Average gait cycle time (CT) of patients without gait difficulty is 1.1 s (SD 0.32), smaller than the average gait cycle time 1.22 s (SD 0.49) of patients having gait difficulty. The stride length and walking speed of patients without gait difficulty are significantly larger than patients with gait difficulty. The average acceleration of walking is 5.2 m/s^2^ (SD 1.1) for patients without gait difficulty, and 3.6 m/s^2^ (SD 1.7) for patients with gait difficulty. There were statistically significant differences between the two groups for SL (*P*=.035), SP (*P*=.026), and AVG_ACC (*P*=.038). In turning, the number of steps to complete turning 360° (NUM_TURN) and turning speed (TURN_SP) were compared between the two groups. Patients without gait difficulty needed 3.1 (SD 0.9) steps for turning a full circle, and their average turning speed was 79.1 degree/s (SD 8.49). For the group of patients with gait difficulty, 5.4 steps (SD 1.1) were needed and turning speed was 53.7 degree/s (SD 6.98). The differences of these two turning features are also statistically significant (*P*=.042 for NUM_TURN and *P*=.039 for TURN_SP).

**Table 5:** Extracted motion features from walking and turning tests (results are divided into two groups, UPDRS^a^gait score <2 and UPDRS gait score ≥2.)

Gait features		UPDRS gait score <2	UPDRS gait score ≥2	*P* value
		Mean (SD)		
**Walking straight**				
	CT(s)^b^	1.1 (0.32)	1.22 (0.49)	.076
	SL(m)^c^	1.26 (0.17)	1.07 (0.29)	.035^h^
	SP(m/s)^d^	1.15 (0.20)	0.87 (0.28)	.026^h^
	AVG_ACC(m/s^2^)^e^	5.2 (1.1)	3.6 (1.7)	.038^h^
**Turning 360** ^o^				
	NUM_TURN^f^	3.1 (0.9)	5.4 (1.1)	.042^h^
	TURN_SP(degree/s)^g^	79.1 (8.49)	53.7 (6.98)	.039^h^

^a^UPDRS: Unified Parkinson’s Disease Rating Scale.

^b^CT: gait cycle time.

^c^SL: stride length.

^d^SP: walking speed.

^e^ACC: average acceleration.

^f^NUM_TURN: the number of step used to finish turning 360^o^.

^g^TURN_SP: the speed of turning 360^o^, calculated by 360^o^ / time.

^h^Significance (α=.05).

### PD Symptoms Detection and Severity Estimation

The two classification models for detecting gait difficulty and hand resting tremor were trained and validated against the experts’ evaluation result by using 5-fold cross-validation (CV) [47]. We randomly split data from the 40 patients into five equal size subsets. A single subset was retained as the validation data for testing the model, and the remaining four subsets were used as training data. The cross-validation process was then repeated five times with each of the five subsets used exactly once as the validation data. The validation results demonstrate good sensitivity and specificity. For hand resting tremor detection, sensitivity was .77 and specificity was .82. In gait difficulty detection, sensitivity was .89 and specificity was .81. The regression models also showed that captured motion features have strong correlation with PD disease stage, hand resting tremor severity, and gait difficulty severity. The correlation coefficients for PD stage are *r*=.81, *r*=.74 for hand resting tremor, and *r*=.79 for gait difficulty.

## Discussion

### Principal Results

Today’s mobile devices empower consumers more than ever to measure, collect, access, and manage health data [48]. As acquisition and processing of large scale data become feasible in the cloud, they have the potential to vastly improve decision making and provide important insights about personal health and public health. In this study, we designed a mobile cloud solution, PD Dr, for Parkinson’s disease home-based monitoring. This paper describes the system architecture, basic functional components, and data flow of the mobile cloud app in remote health monitoring and chronic disease management for patients with PD. This system could be extended to assessment of other movement disorders.

Although home-based monitoring increases health care access and saves patients’ time and money, it also places stringent constraints on system: cost, size, unobtrusiveness, and ease of use are all factors impacting usefulness of the system. System testing by recruited patients showed PD Dr is simple and easy to use. Users can finish all tests within 5 minutes. Another advantage of PD Dr is that it can provide a more objective and quantitative measurement for PD assessment than subjective evaluation by physicians in PD assessment. Results of system evaluation tests demonstrated that PD Dr can effectively capture important motion features from accelerating signals to differentiating tremor and severity of gait impairment. The acceleration amplitude and power spectrum density of hand tremor give more quantitative and objective measurement than subjective rating. Motion features extracted from walking and turning demonstrated intuitive description of the subject’s walking ability, and comparison of walking and turning motion features show significant difference between gait impairment. The predictive models constructed in this study to estimate disease severity demonstrated acceptable accuracy and promising future. The ability to monitor secular changes in tremor amplitude and various components of the gait cycle can provide a powerful tool for the patient and clinician to monitor progression of disease and need to optimize treatment and use services like physical therapy to address incipient problems in gait and postural stability. Further, the modular design of the cloud-based data processing and decision support units allow the ability to process accelerometer-based data acquired using a multitude of wearable devices, which are being introduced in the consumer market at a rapid pace.

Despite many studies and research on ambulatory assessment systems for Parkinson’s disease, most of the previous approaches were based on using a separate sensor network and control module. In Patel’s recent study based on wearable sensors, the user needs to wear eight accelerometers on the body, and requires a mobile phone to receive and send data [8]. In Keijsers’s study on ambulatory motor assessment in PD, they used six sensors at different positions of body to collect data [13]. In the above systems, the sensed data needs to be transmitted to a control device and wearing multiple sensors on the body is not easy or convenient for PD patients. The detached sensor and control device also increase the system complexity and reduce the system reliability. Users need to set up and configure the system properly, which also decreases the usability [44,49]. In PD Dr, no complicated set-up or configuration is needed. Users simply use one smartphone and one strap to finish all tests, with no environment and space limitations. No peripheral equipment, such as sensors, cables, or power supply is needed, which greatly improves the usability of PD Dr. Another advantage of PD Dr is it provides an integrated service from data capturing to symptom detection and severity assessment. More importantly, PD Dr serves as an ambulatory PD evaluation platform; it is easy to add new tests to extend function and measure more PD characteristics by taking advantage of the smartphone sensor and various data input channels. For example, the touch screen can be used for measuring finger tapping speed, and video recording ability can capture the facial expression to evaluate loss of facial expression.

### Limitations

Since this paper mainly focuses on system design, components, and architecture, we did not present details about data processing, motion feature extraction, and decision-making models for severity estimation or symptoms identification. We plan to discuss details about the data pipeline, motion feature extraction, and results of estimating disease severity using machining learning to support decision-making in a subsequent paper.

As a pilot study, this prototype system first focused on tremor and gait assessment in PD. Other than these two key PD characteristics, bradykinesia, motor fluctuation, and dyskinesia are also substantial symptoms that have important clinical meaning and also impair patients’ quality of life. Those three PD characteristics are also measurable by using accelerometers. In the next step, we will extend PD Dr to include more tests to cover broader dimensions of Parkinson’s disease.

Unlike the wearable sensor network, PD Dr is not an ideal platform to provide continuous monitoring over a long period of time, but more focuses on intermittent assessment of key motor issues of PD. This intermittent assessment approach, to some extent, has lower temporal resolution compared to wearable sensor networks, which users can wear for a longer time. Therefore, for some specific PD symptoms, like on-off phenomenon, PD Dr has poor ability to catch the change of motor ability during a certain period of time.

In this initial evaluation, due to funding and time limitations, we recruited only 40 PD patients as test subjects. The small number of test subjects, to some extent, limits training a more accurate decision model, as well as validating the performance of the entire system. In the next step, with more available testing data from recruited subjects, we plan to refine the algorithm of cloud server side data analytics to improve the accuracy of key PD symptoms detection and severity estimation.

Another limitation of our evaluation is the absence of feedback from PD physicians. This limitation will be taken into account in future usability tests. The system is still under development. Some additional functionality, including firing alerts when symptoms become worse, providing distributed access to clinicians for reviewing and accessing data repository, will be implemented in the future work.

## Abbreviations

ACC: average acceleration
CT: gait cycle time
FoG: freezing of gait
NUM_TURN: the number of step used to finish turning 360o
PD: Parkinson’s disease
PSD: power spectrum density
SL: stride length
SP: walking speed
TURN_SP: the speed of turning 360o, calculated by 360o / time
UPDRS: Unified Parkinson’s Disease Rating Scale
